# Reappraisal of Bergmann glial cells as modulators of cerebellar circuit function

**DOI:** 10.3389/fncel.2015.00246

**Published:** 2015-07-02

**Authors:** Chris I. De Zeeuw, Tycho M. Hoogland

**Affiliations:** ^1^Cerebellar Coordination and Cognition, Netherlands Institute for NeuroscienceAmsterdam, Netherlands; ^2^Department of Neuroscience, Erasmus MCRotterdam, Netherlands

**Keywords:** astrocytes, Bergmann glia, cerebellum, cerebellar zone, zebrin, neuron-glia interactions

## Abstract

Just as there is a huge morphological and functional diversity of neuron types specialized for specific aspects of information processing in the brain, astrocytes have equally distinct morphologies and functions that aid optimal functioning of the circuits in which they are embedded. One type of astrocyte, the Bergmann glial cell (BG) of the cerebellum, is a prime example of a highly diversified astrocyte type, the architecture of which is adapted to the cerebellar circuit and facilitates an impressive range of functions that optimize information processing in the adult brain. In this review we expand on the function of the BG in the cerebellum to highlight the importance of astrocytes not only in housekeeping functions, but also in contributing to plasticity and information processing in the cerebellum.

## Introduction

The versatile function of astrocytes is well highlighted by the Bergmann glial cell (BG) of the cerebellum, an astrocyte type that outnumbers the principle neuronal output cell of the cerebellar cortex, the Purkinje cell (PC), roughly eight-fold (Korbo et al., 1993; Reichenbach et al., 1995). BGs are essential for migration and correct layering of granule cells in early cerebellar development (Rakic, 1971), but they remain an integral part of the adult cerebellar circuit, where they subserve an important role in extracellular ion homeostasis (Wang et al., 2012), synapse stability (Iino et al., 2001; Saab et al., 2012), plasticity (Balakrishnan and Bellamy, 2009; Balakrishnan et al., 2014), metabolic function and neuroprotection (Poblete-Naredo et al., 2011; Jakoby et al., 2014). The functional relevance of BGs is also reflected by the expression of cyto-architectural markers that largely overlap with those of the cerebellar zones (Reeber et al., 2014), the canonical computational units of cerebellum (Chambers and Sprague, 1955; Groenewegen and Voogd, 1977; Groenewegen et al., 1979; Oscarsson, 1979; Zhou et al., 2014; De Zeeuw and Ten Brinke, 2015). In the intact brain, activity in BGs as measured via *in vivo* calcium imaging reveal a diverse repertoire of signals, including compartmented signaling in BG processes (Hoogland and Kuhn, 2010; Nimmerjahn et al., 2009), large scale elevations of calcium during behavior that are thought to correlate with changes in blood flow (Nimmerjahn et al., 2009), and more confined radially expanding waves that are increased in awake behaving vs. anesthetized animals (Nimmerjahn et al., 2009) and that increase in frequency with age (Mathiesen et al., 2013). In addition, homeostatic control of calcium by BGs may be important for neuroprotection and oxygen regulation (Mathiesen et al., 2013). Importantly, not only direct optogenetic manipulation of calcium levels in BGs (Sasaki et al., 2012), but also inducible genetic deletion of AMPA receptors in BGs can affect associative motor learning and/or motor performance (Saab et al., 2012). Thus BGs are not just involved in essential housekeeping functions, but may also contribute to information processing in the cerebellum. Here, we provide more details on these diverse functions and propose the hypothesis that BGs are involved in fine-tuning activity in cyto-architecturally distinct cerebellar zones.

## Cerebellar Architecture

In order to understand how BGs are integrated in the cerebellum, a brief introduction to its architecture is required. The basic circuit of the cerebellum is evolutionary conserved across vertebrates—from lampreys (Larsell, 1947) to cetaceans (Hanson et al., 2013)—and confers unique computational properties to enable sensorimotor integration and motor coordination with high temporal precision (Llinás and Sasaki, 1989; Welsh et al., 1995; Kistler and De Zeeuw, 2002). There are two main distinguishing features. One includes a rostral-caudal organization of cyto-architectural and functionally distinct sagittally oriented PC zones with further subdivisions into microzones (Groenewegen and Voogd, 1977; Zhou et al., 2014; Tsutsumi et al., 2015). The PCs provide the output of the cerebellar cortex to the cerebellar nuclei (CN) and thereby exert parcellated control over downstream effectors, which are often recruited sequentially during movements (Welsh et al., 1995; Hoogland et al., 2015). The climbing fibers (CFs), which originate from the inferior olive in the ventral medulla oblongata, project to the rostro-caudal zones of PCs (Sugihara et al., 2007; Brown et al., 2012) where they can trigger complex spikes synchronously to adjust movements (Marshall and Lang, 2009; Ozden et al., 2012; De Gruijl et al., 2014). Another integral feature of cerebellar architecture is the transverse alignment of granule cell parallel fiber axons (PFs), which cross the entire width of a cerebellar folium at right angles to the PC dendrites. The intrinsically generated simple spike firing of PCs is tuned not only by PF input, but also by CF input that can trigger short pauses of simple spike firing and determine the phase of their modulation (Schmolesky et al., 2002). Cerebellar zones are demarcated by preferential expression of select proteins in alternating parasagittal bands. The best-known example is aldolase C, or Zebrin II (Leclerc et al., 1992). Zebrin-positive PCs fire intrinsically at lower frequencies (~60 Hz) than PCs in zebrin-negative zones (~100 Hz) (Zhou et al., 2014) and these zones also appear to respond differentially to sensory input (Tsutsumi et al., 2015; Witter and De Zeeuw, 2015), lending support to the idea that cerebellar zones are basic operational units of cerebellar motor control. Other proteins with expression patterns similar or complementary to Zebrin are e.g., the neuronal calcium sensor protein (NCS-1; Jinno et al., 2003), the excitatory amino acid transporter 4 (EAAT4; Dehnes et al., 1998), heat shock protein HSP25 (Armstrong et al., 2001) and others (Cerminara et al., 2015). CFs projecting to zebrin-positive zones release more glutamate and generate more CS spikelets (Paukert et al., 2010). Moreover, the susceptibility of their PC targets for plasticity may be different as well (Wadiche and Jahr, 2005; Wang et al., 2011), highlighting that zones are functionally demarcated. How BGs are embedded in these demarcated zones and contribute to their function is not yet fully understood.

## BG Structure, Circuit Embedding and Structural Plasticity

**Figure 1:**
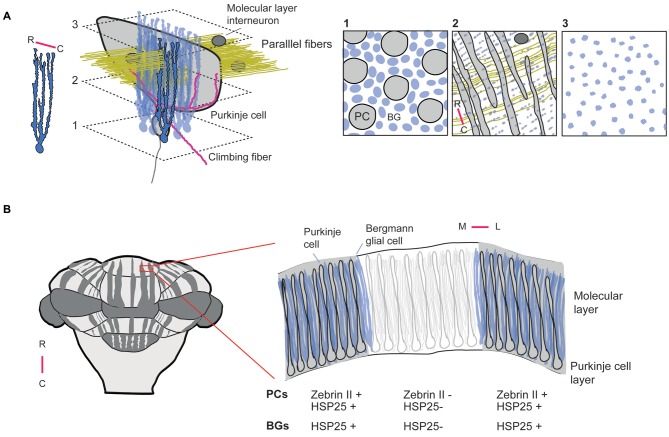
**Embedding of Bergmann glial cells in cerebellar circuits and zones (A).** Arrangement of BGs relative to a single purkinje cell (PC). Left: Single BG indicating that radial fibers predominantly branch in the parasagittal plane, i.e., along the rostro-caudal axis of the cerebellum. BGs have up to 5 radial fibers that extend over the full depth of the molecular layer. In addition, BG radial fibers give off small side branches or BG microdomains (not shown) that predominantly wrap around parallel fiber- PC synapses (Grosche et al., 2002). Right: planes at different depths illustrating the PC-BG relationship. Inset 1: PC layer with arrangement of a monolayer of BG and PC somata at a density of 8 BGs (blue) to 1 PC. Inset 2: molecular layer BG processes arrange in palisades to maximize interaction with parallel fibers. Inset 3: BG fibers terminate at the surface with bulbous end-feet, the function of which has not been studied. **(B)** Zonation in the cerebellum. Left: Zebrin II (zebrin) expression in the cerebellum follows a pattern consisting of rostro-caudal parasagittal stripes that constitute functional modules with distinct input-output relationships. Right: coronal section through a cerebellar folium showing clear delineation of zebrin-positive, or negative borders. Other proteins such as the HSP25 display staining complementary to Zebrin II. BGs display a similar zonal expression of HSP25 (Reeber et al., 2014), but with less distinct boundaries due to their morphology. R: rostral, C: caudal, M: medial, L: lateral.

Several studies have described the cellular and subcellular structure of BGs in detail (de Blas, 1984; Siegel et al., 1991; Reichenbach et al., 1995; Castejón et al., 2002). BGs are distinct in having up to five polarized main processes (radial fibers) that extend over the full depth of the molecular layer. The BG fibers branch in the parasagittal plane (Figure 1), but overlap with fibers of neighboring cells to form palisades. In rodents BG fibers are regularly spaced at intervals of a few μm oriented along the parallel fiber direction with a bit wider spacing along the rostro-caudal axis of the cerebellum (de Blas, 1984; Reichenbach et al., 1995; Hoogland and Kuhn, 2010). BG radial fibers give rise to small convoluted side branches that form sites of putative neural-glial interaction and account in rat for 90% of the BG membrane surface area (Grosche et al., 1999, 2002). Two classes of protrusions from the main BG fibers have been distinguished, short thorny processes and more elaborate processes with long thin stalks several μm long. The complex BG appendages form microdomains that have surface-to-volume ratios six-fold higher than the main radial fibers, show highly complex branching patterns, and by nature of their structure can act as electrotonically and biochemically compartmentalized microdomains subserving on average five synapses (Grosche et al., 1999). Estimates on the number of PC synapses that BG microdomains encompass ranges from ~2000–6000 (Reichenbach et al., 1995). The ensheathment of PC synapses commences near onset of synaptogenesis and could be important for regulation of synapse number, though it does not affect synapse stability in adulthood (Lippman Bell et al., 2010). What other functions do BG microdomains have? One possibility is that they restrict diffusion of neurotransmitter from the synaptic cleft (Grosche et al., 2002) and thus help to improve the fidelity of synaptic transmission. Due to the arrangement of BG palisades, side processes are ideally positioned to sample and interact with PFs (Herndon, 1964). High immuno-reactivity for glutamine synthetase was found in BG processes (Reichenbach et al., 1995) and BGs express high densities of glutamate transporters (Storck et al., 1992; Rothstein et al., 1994; Bergles et al., 1997). Together, this implicates that BGs may play a role in PF-mediated synaptic transmission. Strikingly, the first postnatal weeks show an impressive elaboration of BG appendages that parallels the development of PFs (Shiga et al., 1983; Grosche et al., 2002). Electron microscopy data have demonstrated that BG processes not only enwrap PF–PC synapses, but also appose PFs, CF collaterals, and processes of molecular layer interneurons (MLIs; Castejón et al., 2002). BG processes also express GABA_A_ receptors in the vicinity of inhibitory synapses close to PC somata, but in a small fraction also at excitatory synapses near PC dendritic spines (Riquelme et al., 2002). Thus, BGs are equipped to sense both inhibitory and excitatory neurotransmitters. Indeed, glutamate transporters are densely expressed on BG processes and can aid glutamate uptake into BGs (Bergles et al., 1997). It has been demonstrated that GLAST and GLT-1 also affect the time course of synaptically evoked currents during repeated PF activation of a few fibers, or during single stimuli when multiple nearby fibers are activated (Marcaggi et al., 2003). By shaping the time course of postsynaptic currents glutamate transporters in BGs could regulate mGluR—mediated plasticity (Marcaggi and Attwell, 2005). Both electrical stimulation of PFs and ATP release from MLIs can trigger calcium elevations in BG processes (Beierlein and Regehr, 2006). BGs normally express calcium-permeable α-amino-3-hydroxy-5-methyl-4-isoxazolepropionic acid (AMPA) receptors, as demonstrated by the presence of glutamate evoked AMPAR currents and immunocytochemistry (Burnashev et al., 1992; Sato et al., 1993; Bellamy and Ogden, 2006; Saab et al., 2012). They are required for maintenance of BG processes around PC dendritic spines (Iino et al., 2001). Recent work in which (calcium-permeable) AMPA receptors (GluR1 and GluR4) were conditionally knocked-out in BGs has replicated this finding and demonstrated a co-occurrence of such BG process retraction with impairments in associative motor learning during both the ErasmusLadder task and eyeblink conditioning (Saab et al., 2012). Moreover, since these phenotypes occurred in adult but not young animals, the picture emerges that BGs also serve specific and active functions in adulthood. Interestingly, AMPA receptors probably also regulate electrical coupling between BGs, as their activation results in a strong reduction of BG-BG junctional conductance (Müller et al., 1996). Even though the BG palisades are oriented along the direction of the PFs, the electrical coupling between BGs appears to be limited along the parasagittal plane matching the orthogonal orientation of PC dendritic arbors (Figure 1A).

## Types of Calcium Signaling in BGs

Calcium dynamics in BGs have been described in detail *in vitro* and *in vivo*. Burst stimulation of PFs is sufficient to trigger local calcium increases in BG processes (Grosche et al., 1999; Beierlein and Regehr, 2006; Piet and Jahr, 2007). Such calcium elevations are sensitive to block of group I metabotropic glutamate receptors, purinergic (P2Y) receptors or AMPAR currents (Beierlein and Regehr, 2006; Piet and Jahr, 2007). Putative AMPAR-mediated calcium increases show shorter duration and smaller amplitude calcium transients upon stimulation, while larger calcium increases are sensitive to cyclopiazonic acid (CPA), blocking release from calcium stores. The slow calcium transients seen during burst stimulation can be elicited by ATP released from MLIs upon PF stimulation (Piet and Jahr, 2007). Stimulation of CFs does not elicit calcium elevations in BGs, but does evoke currents in BGs. These currents are of much lower magnitude than during PF stimulation. PF stimulation results in BG AMPAR currents that display paired pulse facilitation (PPF) as seen with synaptic PPF, but such AMPAR-mediated BG currents are of short duration as BG AMPAR currents with high-frequency stimulation cannot be sustained (Bellamy and Ogden, 2005). Taken together, it appears that BGs are more responsive to PF than to CF activity. However, synchronous CF activity could in principle boost glutamate spillover. Spillover from CF terminals has been associated with larger CF mediated AMPAR currents and could act as a cue to guide BG processes to enwrap synapses for better isolation (Matsui and Jahr, 2004). Thus, functionally BG AMPAR currents appear to be important for stabilizing BG microdomains around synaptic elements to enable effective clearance through BG glutamate transporters (Rothstein et al., 1994; Bergles et al., 1997) and thereby increase the fidelity of synaptic transmission.

Electrical stimulation of PF axonal beams in recent experiments have demonstrated clustered activation of PFs in response to sensory stimulation (Wilms and Häusser, 2015). Such PF activation could result in calcium elevations in BG processes *in vivo* similar to those that have been reported *in vitro*. In vivo, several types of BG calcium responses have been observed. These include for example single process calcium elevations as revealed through sparse viral transduction of BGs with a genetically encoded calcium indicator (GECI; Hoogland et al., 2009) or synthetic calcium indicators in combination with the astrocyte marker SR101 (Nimmerjahn et al., 2004). In addition, elevations in large fields of BG processes can occur *in vivo* during locomotion (Nimmerjahn et al., 2009; Paukert et al., 2014) and/or transglial calcium waves (Hoogland et al., 2009; Nimmerjahn et al., 2009). The frequency of BG signals of mammals in the awake state is significantly higher than those in the anesthetized state (Nimmerjahn et al., 2009; Hoogland and Kuhn, 2010) and their rate is generally sensitive to block of neural activity and glutamatergic transmission with some remaining calcium responses that might be intrinsically generated (Nimmerjahn et al., 2009). The calcium elevations in BG processes during locomotion probably reflect the increased synaptic drive observed in the cerebellum during locomotion (Ozden et al., 2012). However, it should be noted that the correlations with the onset of locomotion are weak and that BGs do not always respond with calcium increases during bouts of locomotion (Paukert et al., 2014). Transglial calcium waves in the cerebellum are triggered by ATP and rely on release of calcium from internal stores (Hoogland and Kuhn, 2010). Their frequency also increases when the animal is transferred from an anesthetized to an awake state (Nimmerjahn et al., 2009) or when it is getting older (Mathiesen et al., 2013). Indeed, the role of these waves may be metabolic and neuroprotective in that their occurrence increases with low oxygen tension (Mathiesen et al., 2013). Future studies employing selective expression of GECIs should further elucidate how calcium microdomains in BGs respond to sensorimotor stimulation in detail (Kuhn et al., 2011; Paukert et al., 2014).

## BG K^+^ Siphoning and Functional Implications

Astrocytes including BGs act as large sinks for redistribution of ionic gradients and could thereby have significant impact on neurotransmission and excitability of neurons (Newman et al., 1984; Reichenbach et al., 1995). This can have clear benefits for redistributing ions in regions where K^+^ accumulates rapidly during periods of strong activity, ensuring that increased neuronal activity can be sustained. In fact, large 1–3 mM accumulations of extracellular K^+^ have been measured *in vitro* around PCs in response to spiking activity (Hounsgaard and Nicholson, 1983). The presence of inward rectifying K ^+^ (KIR) channels can effectively shuttle K^+^ into BGs (Butt and Kalsi, 2006), which have strongly hyperpolarized resting membrane potentials. *In situ* hybridization studies have shown that KIR4.1 has highest expression levels in hippocampus and cerebellar cortex (Poopalasundaram et al., 2000). In the cerebellum KIR4.1 channels are expressed in BGs near the PC primary branches (Poopalasundaram et al., 2000). Recent work has revealed the importance of BGs in regulating K^+^ concentrations to influence PC membrane potential. Such modulation was shown to be Ca^2+^-dependent and could trigger bistability of PCs (Wang et al., 2012), a phenomenon in which PCs show periods of firing alternated by quiescence. In the intact animal PC bistability has been shown to be strongly influenced by anesthetics and mostly absent in healthy tissue of awake animals (Schonewille et al., 2006), but it could potentially be present in the cerebellum during sleep, which currently is an active area of investigation. The function of bistability is contentious, but modeling studies suggest that it can increase the capacity of PCs to learn input-output associations (Clopath et al., 2012). Thus, since artificial elevation of calcium in BGs *in vitro* transiently reduces extracellular K^+^ concentrations (Wang et al., 2012), the question emerges to what extent a particular type of calcium signal is preferably driving inward K^+^ currents in BGs *in vivo* or whether other observed types of calcium conductances also contribute to regulation of K^+^ in the intact cerebellum. While ATP-driven calcium elevations could trigger transient shifts of PCs to an upstate *in vitro*, ATP-mediated transglial waves relying on calcium release from intracellular stores could in principle modulate excitability of nearby PCs *in vivo* (Hoogland et al., 2009). However, no direct role of transglial waves in physiological function has yet been demonstrated. As it stands now, these signals seem to be involved predominantly in metabolic control or are neuroprotective (Mathiesen et al., 2013). As mentioned above, the frequency of transglial waves is significantly decreased under anesthesia (~7-fold) when bistability manifests itself most prominently (Nimmerjahn et al., 2009). This could be reconciled by the fact that increased frequency of BG calcium signals in awake mice (Nimmerjahn et al., 2009; Hoogland and Kuhn, 2010) could cause a constitutive reduction of external K^+^ to drive PCs into prolonged (depolarized) up-states. It has been proposed that PCs remain in the upstate because calcium-dependent K^+^ channels gradually inactivate after hyperpolarization (Wang et al., 2012), but the exact mechanisms are still unclear. Both locally and globally BGs are able to alter excitability by regulating extracellular K ^+^ but under which conditions this happens in the intact brain requires further investigation.

## Activation of BGs during Vigilance

BGs like other astrocytes express receptors for the neurotransmitter noradrenaline and activation of such receptors can trigger calcium elevations (Salm and McCarthy, 1990; Kirischuk et al., 1996). Recent studies have revealed that noradrenaline-dependent calcium signaling in astrocytes can be triggered *in vivo* (Bekar et al., 2008; Ding et al., 2013), either after stimulating the source of noradrenergic afferents, the locus coeruleus, after strong peripheral stimulation, such as foot shocks, or even with whisker stimulation as revealed by pharmacological block with alpha-adrenergic receptor antagonists (Bekar et al., 2008; Ding et al., 2013). The release of noradrenaline is non-synaptic, diffuses throughout the neuropil volume and can thereby trigger calcium elevations and down-stream signaling in astrocytes.

Possibly, noradrenaline release also contributes to BG activation during sensorimotor stimulation. Indeed during head-fixed treadmill locomotion (Nimmerjahn et al., 2009) weak responses can be seen in BGs of the cerebellum across the field of view. These signals can be extracted from ROIs defined by the co-loading of the astrocyte marker SR101 (Nimmerjahn et al., 2004)—now known to also affect neuronal excitability (Kang et al., 2010)—and OGB-1/AM, a synthetic calcium indicator dye. Calcium signals in (purported) BG processes occur during locomotion bouts and are attenuated with short inter-movement intervals, suggesting that calcium release from internal stores underlies the calcium increases. In a recent study in which the GECI GCaMP3 was expressed selectively in astrocytes, calcium elevations were also demonstrated to encompass large fields of BG processes during locomotion bouts, but they were of low amplitude and such events had a failed rate of about one in three (Paukert et al., 2014). Strikingly, when locomotion was enforced (and thus arousal peaked) significantly larger whole field BG responses were evoked in a consistent manner. These also appeared refractory. Using pharmacological tools it was subsequently shown that such arousal-induced BG calcium elevations were dependent on noradrenaline release and activation of the α1-adrenergic receptor similar to what has been observed for cortical astrocytes (Ding et al., 2013). Calcium elevations were not restricted to BGs, but also observed simultaneously in visual cortex astrocytes—albeit with a slightly longer delay—suggesting that release of noradrenaline during a state of arousal activates astrocytes across the entire brain. Thus, the noradrenergic system can act as a gain modulator not only for neurons (Johnson et al., 1968), but also astrocytes during active behavior. Which types of natural behaviors trigger the arousal system to elicit calcium elevations in BGs remains to be elucidated.

## BGs and Cerebellar Zone Patterning

The modular organization of the cerebellum into parasagittal zones as defined e.g., by Zebrin, or other markers (Figure 1B) has been known for decades (Voogd, 1969; Groenewegen et al., 1979), and although hypotheses were formulated on their function (Oscarsson, 1979), it was not until much later that their functional organization was investigated in awake animals (Welsh et al., 1995; Lang et al., 1999; Ozden et al., 2012; De Gruijl et al., 2014). *In vivo* two-photon microscopy recently revealed that zebrin-positive zones not only define sharp anatomical borders, but also show a sharp delineation of functional responses to sensory stimuli (Tsutsumi et al., 2015). Although it is logical to assume that BGs embedded in cerebellar zones follow the same modular organization as the neurons they interact with, it was not until recently that evidence was presented that BGs indeed show overlap with cerebellar zones (Reeber et al., 2014), as defined by marker proteins also found in PCs. Specifically, it concerned the heat shock protein, HSP25, which was shown earlier to be confined to zebrin-like parasagittal bands of PCs in mice (Armstrong et al., 2000). The function of this protein in the cerebellum is still unknown. BG processes do not have the flat topography of PC dendritic arbors and the BG borders defined by HSP25 are not as tight as those seen for PCs (Figure 1B). Nevertheless, there is substantial overlap. It is likely that other zone-delimiting proteins will be found to co-localize not only with cerebellar neurons, but also BGs. Interesting as this may be, the most important question remains to be answered, namely do BGs that are integrated in cerebellar zones also contribute actively to the physiology of these zones? The recent establishment of differential firing behavior of PCs in zebrin-positive vs. zebrin-negative cerebellar zones—with the latter firing at higher frequencies—(Zhou et al., 2014) has made this question quite relevant. It was found that the higher frequency of PC firing in zebrin-negative zones could be attributed to the activation of the TRPC3 channel in PCs, a channel that is under control of proteins that have expression patterns overlapping with zebrin-negative zones. Although the firing rate differences were strongly attenuated when blocking TRPC3 channel function, alternate pathways are feasible through which PCs activity levels could be set in cerebellar zones. BGs have been shown to tonically release GABA via bestrophin 1 (Best1) channels (Lee et al., 2010; Yoon et al., 2011). The close apposition of BG processes to PFs, PCs and MLIs suggest that through tonic release of neurotransmitters BGs are capable to set activity levels in the cerebellar circuit. In granule cells tonic GABA release from astrocytes through Best1 could evoke tonic currents of up to ~30 pA. In BGs the concentration of GABA has been estimated with the use of immunogold labeling to be between 5–10 mM and is synthesized by monoamine oxidase (Yoon et al., 2014). Thus a sufficient electrochemical gradient exists to drive currents through Best1 in BGs and sustain tonic GABA release. The GABAB receptor 2 (GABABr2) is found in PCs of zones that label positively for zebrin (Chung et al., 2008). Given the typical extrasynaptic localization of such receptors (Fritschy et al., 1999) and their known coupling to G protein-coupled inwardly-rectifying potassium (GIRK) channels that can drive membrane hyperpolarization (Lüscher and Slesinger, 2010), it is tempting to speculate that tonic GABA release in Zebrin/GABABr2-positive zones sets, or maintains lower activity levels. In addition, the exact distribution of Best1 in the cerebellum has not been reported in great detail as histology was performed on thin parasagittal sections, making it hard to assess whether perhaps Best1 itself is also expressed preferentially in a zebrin-like pattern (Lee et al., 2010; Yoon et al., 2011). Regardless, tonic GABA release from BGs (in combination with zone-delimited expression of GABA receptors) adds another layer of glial control over cerebellar circuit function over time courses that exceed the typical time scale of seconds, during which BGs modulate their activity.

## BGs and Information Processing in the Cerebellum: Future Challenges

BG are versatile in their function and highly integrated in the cerebellar circuit. However, many questions remain about their exact contribution to information processing in the cerebellum. High surface-to-volume ratios and thin stalks endow BG microdomains (Grosche et al., 1999) with the ability to act as independent compartmentalized units that aid in glutamate uptake (Bergles et al., 1997), shaping of fast and slow synaptic currents (Marcaggi et al., 2003; Marcaggi and Attwell, 2005), regulation of extracellular K^+^ (Wang et al., 2012) and tonic GABA release (Lee et al., 2010). Furthermore, their activity is co-modulated with neurons during increased states of vigilance (Paukert et al., 2014). Thus at a local scale BGs can modulate the efficacy of synaptic transmission of a cluster or even individual synapses, possibly facilitating memory formation associated with specific behaviorally relevant contexts. The co-expression with PCs of zone-delimiting proteins suggest that BGs in cerebellar zones could either sustain reported physiological differences in firing behavior of neurons in such zones, or set their activity levels. Concurrent electrophysiological targeting of BGs and nearby neurons and *post hoc* immunohistochemistry for zone-delimiting proteins, or transgenic mouse models that allow direct visual selection of BGs in cerebellar zones should advance our knowledge in this regard. A combination of targeted electrophysiology (Kitamura et al., 2008) and cell-selective expression of GECIs in BGs (Chen et al., 2013) should allow better assessment of the relation between calcium dynamics in BG fibers and microdomains and their temporal relation with sub- and supra-threshold activity in the neurons they interact with.

## Conflict of Interest Statement

The authors declare that the research was conducted in the absence of any commercial or financial relationships that could be construed as a potential conflict of interest.
